# Carbapenem-Resistant, Virulence Plasmid–Harboring *Klebsiella pneumoniae,* United States

**DOI:** 10.3201/eid3104.241396

**Published:** 2025-04

**Authors:** Jianping Jiang, Tengfei Long, Adeline R. Porter, Arianne Lovey, Annie Lee, Jesse Thomas Jacob, Cesar A. Arias, Robert Bonomo, Robert Kalayjian, Yanan Zhao, Frank R. DeLeo, David van Duin, Barry N. Kreiswirth, Liang Chen

**Affiliations:** Hackensack-Meridian Health Center for Discovery and Innovation, Nutley, New Jersey, USA (J. Jiang, T. Long, A. Lovey, A. Lee, Y. Zhao, B.N. Kreiswirth, L. Chen); National Institute of Allergy and Infectious Diseases Rocky Mountain Laboratories, Hamilton, Montana, USA (A.R. Porter, F.R. DeLeo); Emory University, Atlanta, Georgia, USA (J.T. Jacob); Houston Methodist Hospital and Houston Methodist Research Institute, Houston, Texas, USA (C.A. Arias); Louis Stokes Cleveland VA Medical Center, Cleveland, Ohio, USA (R.A. Bonomo); Case Western Reserve University School of Medicine, Cleveland (R.A. Bonomo); MetroHealth Medical Center, Cleveland (R. Kalayjian); University of North Carolina at Chapel Hill, Chapel Hill, North Carolina, USA (D. van Duin)

**Keywords:** *Klebsiella pneumoniae*, carbapenem resistance, bacteria, virulence plasmid, pVir-CRKP, virulence, clinical and genomic characterization, antimicrobial resistance, United States

## Abstract

Carbapenem-resistant and virulence plasmid–harboring *Klebsiella pneumoniae* (pVir-CRKP) has emerged and spread globally, yet clinical investigations from the United States remain limited. We conducted a genomic analysis of 884 unique carbapenem-resistant *K. pneumoniae* isolates from a multicenter US cohort and identified 6 pVir-CRKP isolates, including 2 sequence type (ST) 23, 2 ST893, and 2 ST11 isolates. Patients infected with pVir-CRKP experienced high Pitt bacteremia scores and a 33% 30-day mortality rate. The pVir-CRKP isolates exhibited significant sequence variation in virulence genes and plasmids, along with differences in mucoviscosity, capsule production, survival in normal human serum, resistance to killing by human polymorphonuclear neutrophils, and in vivo pathogenicity. Phylogenetic analyses showed that most pVir-CRKP isolates were genetically similar to strains reported from other global regions. The emergence of pVir-CRKP with higher virulence potential and carbapenem resistance in the United States than the predominant carbapenem-resistant *K. pneumoniae* clone underscores the need for active global surveillance.

Carbapenem-resistant *Klebsiella pneumoniae* (CRKP) is a major concern to global public health because it is highly resistant to most β-lactam and β-lactamase inhibitors (*1*). Strains termed hypervirulent *K. pneumoniae* (HvKP) cause community-acquired invasive infections in healthy persons (*2*). The emergence of convergent strains of *K. pneumoniae*, possessing features of both HvKP and CRKP, has elevated the concern by linking virulence and resistance in a single strain (*3*). The World Health Organization has warned of a global increase in both HvKP and CRKP (*4*).

The convergent strains are reported mostly from East Asia but also from other global regions. Several previous studies documented the acquisition of carbapenemase-encoding plasmids in HvKP strains. Karlson et al. (*5*) reported a *bla*_KPC-2_–carrying sequence type (ST) 23 HvKP isolate from the United States, and Chen et al. (*6*) reported 18 *bla*_KPC-2_–carrying ST23, ST65, and ST86 HvKP isolates from Singapore. In addition, Beyrouthy et al. (*7*) reported a *bla*_OXA-48_–harboring ST86 HvKP isolate from France. The movement of virulence plasmids into carbapenem-resistant strains has also been reported. In 2018, Gu et al. (*8*) identified 5 virulence plasmid–harboring ST11 CRKP strains from China with enhanced virulence (compared with strains lacking the plasmid); more recently, Zhou et al. (*9*) found that a large proportion of ST11 CRKP strains from China have acquired virulence genes or plasmids. 

The virulence of HvKP isolates is attributed mainly to a pK2044-like plasmid (pVir) that harbors genes encoding mucoid regulators and siderophores (*10*). Mucoid regulators, including RmpADC and RmpA2, enhance capsule production and mucoviscosity of HvKP strains, increasing resistance to neutrophil phagocytosis and serum complement-mediated killing (*11*). Siderophores, which are small molecules secreted by bacteria to accumulate iron, include pVir-encoded aerobactin (*iuc*) and salmochelin (*iro*) (*12*), as well as 2 chromosome-encoded siderophores, yersiniabactin (*ybt*) and colibactin (*clb*), which also contribute to virulence (*12*). Nevertheless, genotypic prediction of HvKP virulence remains a challenge because the presence of virulence genes does not always correlate with increased virulence in vivo. Russo et al. indicated that the combination of 5 pVir-borne virulence factors, namely *iucA* (from the *iuc* gene cluster), *iroB* (from the *iro* gene cluster), *peg-344* (a metabolite transporter), *rmpA* (from the *rmpADC* gene cluster), and *rmpA2*, had an overall accuracy of 94% in predicting the hypervirulence phenotype of HvKP in an animal infection model (*13*).

In this study, we analyzed the genomic features of carbapenem-resistant and virulence plasmid–harboring *K. pneumoniae* (pVir-CRKP) isolates from US cohorts associated with the international multicenter prospective Consortium on Resistance Against Carbapenem in *Klebsiella pneumoniae* and other Enterobacterales 2 (CRACKLE-2) study (*14*,*15*). We describe the clinical characteristics of pVir-CRKP isolates and conducted a comprehensive phenotypic analysis to assess their in vitro, ex vivo, and in vivo virulence, and tracked the phylogenetic origins of those pVir-CRKP strains. 

## Materials and Methods

### Study Cohort

CRACKLE-2 (ClinicalTrials.gov no. NCT03646227) consecutively enrolled hospitalized patients with carbapenem-resistant Enterobacterales (CRE) isolates from all anatomic sources during 2016–2018 (*14*,*15*). In our analysis, patients from the United States were eligible for inclusion when their first qualifying CRE culture was positive for *K. pneumoniae*. The Institutional Review Board of each study site approved the study. We evaluated Charlson comorbidity index (CCI) (*16*), Pitt bacteremia score (*17*), and desirability of outcome ranking (DOOR) (*14*) as previously described. 

### Quantifying Mucoviscosity and Capsule Production

We measured mucoviscosity as previously described (*18*). In brief, we adjusted overnight cultures to a 600-nm optical density (OD_600_) of 1 and then centrifuged. We measured the OD_600_ values (hypermucoid index) in 3 samples. We quantified the capsule using the uronic acid method, as previously described (*19*) (Appendix) 

### Whole-Genome Sequencing and Bioinformatics Analysis

We performed whole-genome sequencing (WGS) as previously described using an Illumina NextSeq 500 platform (https://www.illumina.com) with 2 × 150 bp paired-end reads (*15*). We assembled draft genomes using SPAdes version 3.13.0 as described (*20*). We used Kleborate version 2.3 (*21*) to analyze sequence type, capsular type, *rmpADC*, *rmpA2*, *ybt*, *clb*, *iuc*, and *iro*. We detected *peg-344* by BLASTn version 2.16.0 (ftp://ftp.ncbi.nlm.nih.gov/blast/executables/blast+) against the gene sequence (GenBank accession no. BAH65947.1). We identified resistance genes by AMRFinderPlus version 3.10.20 (*22*), and ARIBA version 2.14.6 (*23*). We considered isolates with contig sequences showing >95% BLASTn identity and >65% sequence coverage to pK2044 (accession no. AP006726.1), and harboring *iuc*/*iro* and *rmpADC*/*A2* as pVir-KP. We sequenced those pVir-KP isolates with Oxford Nanopore (https://nanoporetech.com) and hybrid assembled them with short reads to closure via Unicycler version 0.4.9 (*24*). We visualized linear alignment using Easyfig version 2.1 (*25*) and used Prism 10 for Windows 64-bit version 10.2.3 (GraphPad Software, https://www.graphpad.com) for data analysis.

### Serum Bactericidal Activity and Polymorphonuclear Neutrophil Phagocytosis Assays

We determined serum bactericidal activity as described (*26*). In brief, we combined 5 × 10^5^ colony-forming units (CFU) of bacteria with normal human serum (NHS) and RPMI/H medium in an assay. We rotated assay tubes, diluted serially, and plated on LB agar plates. We calculated percentage survival as CFU_with NHS_/CFU_without NHS_ × 100. We isolated human polymorphonuclear neutrophils (PMNs) using a standard method (*27*) and determined PMN killing as previously described (*26*). We combined, incubated, and diluted bacteria (2.5 × 10^6^ CFU), PMNs (5 × 10^5^ CFU in RPMI/H), and NHS, then plated them on LB agar and enumerated colonies the next day (Appendix). We obtained blood from healthy participants in accordance with a protocol approved by the Institutional Review Board for human subjects at the US National Institutes of Health (protocol no. 01IN055).

### Animal Infection Models

We used a well-established murine intranasal infection model for this study (*28*). We anesthetized female BALB/c mice by intraperitoneal injection. We placed a total of 50 μL of *K. pneumoniae* suspension (≈10^4^ CFUs) on the nares of mice (25 μL per nare) for aspiration into the lungs. We quantified bacterial burdens of each tissue by serial dilutions on LB agar plates (Appendix). We used *K. pneumoniae* strains ATCC43816 (ST493/capsular locus [KL] 2) and NJST258_2 (ST258/KL107) as controls for comparison. Hackensack Meridian Health Institutional Animal Care and Use Committee approved all vertebrate animal experiments.

## Results

### Carbapenem-Resistant *K. pneumoniae* Isolates 

**Figure 1:**
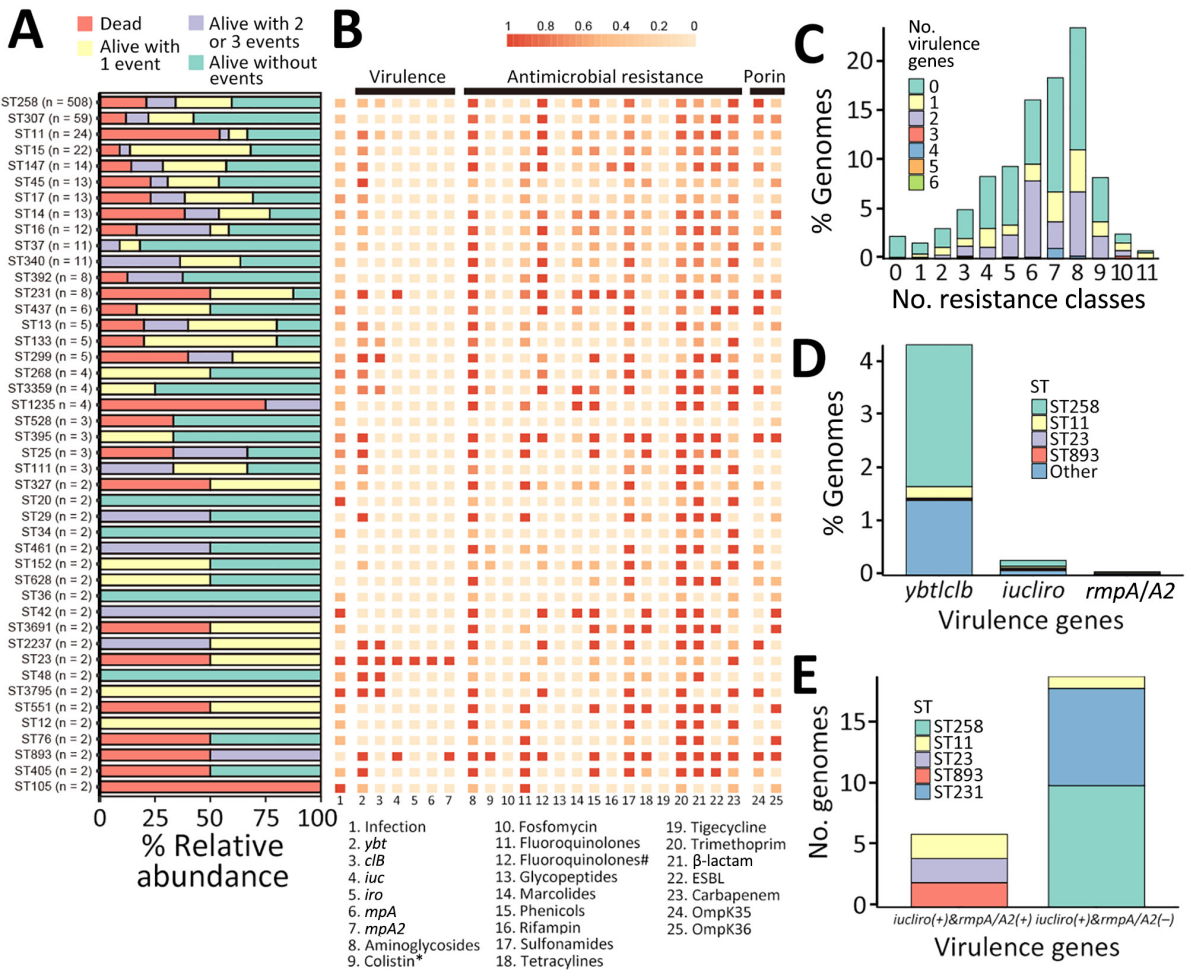
Information of 884 isolates of carbapenem-resistant *Klebsiella pneumoniae* isolates*,* United States. A) Unadjusted distribution of desirability of outcome ranking outcomes. B) Heatmap of infections, virulence genes, antimicrobial resistance genes, and porin mutations in STs with >2 isolates. *MgrB or PmrB loss-of-function mutations. #Mutations in the quinolone resistance–determining regions of GyrA and ParC. C) Distribution of drug classes by genome for which antimicrobial resistance elements were detected. Bars colors indicate the presence of virulence genes. D, E) Distributions of virulence genes (D) and virulence gene combination (E) per genomes. Bars are colored based on STs. ESBL, extended-spectrum β-lactamase; ST, sequence type.

We illustrated DOOR outcomes (Figure 1, panel A), along with the virulome, resistome, and porin mutations associated with different STs (Figure 1, panel B), for 884 CRKP isolates. Among them, 385 (43.6%) isolates possessed antimicrobial resistance genes and >1 of *ybt* (n = 384), *clb* (n = 224), *iuc* (n = 25), *iro* (n = 13), *rmpA* (n = 4), *rmpA2* (n = 5), or *peg-344* (n = 4) genes. CRKP isolates harboring 2 virulence genes (*ybt* and *clb*) and genes encoding resistance to aminoglycosides, fluoroquinolones, sulfonamides, trimethoprim, sulfamethoxazole, β-lactam and carbapenem were the most common (n = 56) (Figure 1, panel C). ST258 isolates with chromosomal *ybt*, *clb*, or both were the most common (237/385 [61.6%]) among virulence gene–harboring CRKP isolates (Figure 1, panel D). For 25 CRKP isolates with >1 of the 5 pVir-borne virulence genes used to predict HvKP in the Russo et al. study (*13*), ST258 (n = 10) and ST231 (n = 8) with *iuc/iro* were the most common. However, only 6 isolates displaying >95% BLASTn identity and >65% sequence coverage compared with pK2044 were pVir-CRKP strains; 2 were ST23, 2 ST11, and 2 ST893 isolates (Figure 1, panel E). ST23 and ST11 isolates harbored all 5 virulence markers (*13*); however, many of those operons and genes were not intact. The remaining 19 CRKP isolates contained plasmidborne *iuc* but showed low coverage (<8%) compared with pK2044 and were negative for *rmpA*/*A2*. Of those 19 isolates, 8 (>75%) displayed high coverage compared with the *iuc-*harboring IncFII(pAMA1167-NDM-5) plasmid pC346_2 (GenBank accession no. CP067712.1) from *K. pneumoniae*, whereas the others exhibited high coverage (> 75%) compared with the *iuc-*harboring IncFIB-IncFIC (FII) plasmid p3PCN033 (GenBank accession no. CP006635.1) from *E. coli* (*29*). In addition, all 10 ST258 strains harbored the *iro* operon in an ICE*Kp10* element on the chromosome.

### Clinical Characterization of pVir-CRKP–Infected Patients

We cultured the 6 US pVir-CRKP isolates from respiratory (n = 3), urine (n = 2), or wound (n = 1) specimens from patients in Cleveland (n = 1) and Houston (n = 1) in 2016 and in Atlanta (n = 4) during 2016 and 2017. Four pVir-CRKP isolates were hospital acquired, and 2 were community acquired. Apart from *K. pneumoniae*, no other pathogens were cultured from the clinical specimens. Five of the patients were middle-aged (median age 56 [IQR 55–60] years), and 5 were male. Of note, patients infected with pVir-CRKP had lower CCI scores (median 2 [IQR 1–4] vs. median 3 [IQR 1–5]) but higher Pitt bacteremia scores (median 3.5 [IQR 2–6] vs. median 3 [IQR 1–5]) compared with patients infected with CRKP (*15*), which suggests that pVir-CRKP isolates possess increased virulence. Patient cases associated with pVir-CRKP demonstrated a 30-day mortality rate of 33%. In contrast, in all the US patients with CRKP infections, unadjusted 30-day all-cause mortality rate was 23% (*15*). However, the small sample size of pVir-CRKP–infected patients may limit the reliability of the observed mortality rate. Two patients with CCI scores >3 died, whereas patients with CCI scores <3 survived without adverse events (Table 1).

**Table 1:** Characteristics of case-patients in study of carbapenem-resistant, virulence plasmid–harboring *Klebsiella pneumoniae* isolates*,* United States*

Patient characteristic	Isolate					
	ARLG-4622	ARLG-3254	ARLG-4744	ARLG-7683	ARLG-3484	ARLG-4720
Location	Cleveland, OH	Houston, TX	Atlanta, GA	Atlanta, GA	Atlanta, GA	Atlanta, GA
Hospital	Hospital A	Hospital B	Hospital C	Hospital C	Hospital C	Hospital C
Department	Emergency department	Medical ward	Medical ward	Surgery ward	ICU	ICU
Collection date	2016 Jun	2016 Sep	2017 Jul	2017 Oct	2016 Dec	2017 Mar
Age group at culture, y	39<49	49<59	49<59	59<69	69<79	49<59
Sex	F	M	M	M	M	M
Hospital or community acquired	Hospital	Community	Hospital	Community	Hospital	Hospital
Culture source	Urine	Respiratory	Urine	Wound	Respiratory	Respiratory
Other pathogens from same source	None	None	None	None	None	None
Infection vs. colonization	Infection	Infection	Infection	Colonization	Colonization	Colonization
Charlson Comorbidity Index score	0	3	4	1	8	1
Pitt bacteremia score	0	3	8	2	6	4
DOOR outcomes	Alive without events	Dead	Dead	Alive without events	Alive with 2 or 3 events	Alive without events

*DOOR, desirability of outcome ranking; ICU, intensive care unit.

### Genomic and Microbiological Characterization of pVir-CRKP 

Genomic analysis classified ARLG-4622 and ARLG-3254 as ST23 with capsule type KL1 and lipopolysaccharides type O1, ARLG-4744 and ARLG-7683 as ST11 with KL64 and O1, and ARLG-3484 and ARGL-4720 as ST893 with KL20 and O3. Capsule polysaccharide (CPS) synthesis genes UDP-glucose dehydrogenase (*ugd*) and acid phosphatase (*cpsACP*) were truncated by frameshift mutations, *ugd* in ARLG-4622 and *cpsACP* in ARLG-3254 (Table 2).

**Table 2:** Genomic features of isolates in study of carbapenem-resistant, virulence plasmid–harboring *Klebsiella pneumoniae* isolates*,* United States*

Genomic feature	Isolate					
	ARLG-4622	ARLG-3254	ARLG-4744	ARLG-7683	ARLG-3484	ARLG-4720
Genome size, Mb	5.6	5.8	5.9	5.9	5.8	5.8
Plasmid number	3	3	5	5	5	5
MLST	ST23	ST23	ST11	ST11	ST893	ST893
K locus type	KL1	KL1	KL64	KL64	KL20	KL20
O locus type	O1/O2v2	O1/O2v2	O1/O2v1	O1/O2v1	O3/O3a	O3/O3a
ESBL or carbapenemase plasmids						
ESBL or carbapenemase	KPC-2	KPC-2	CTX-M-65	CTX-M-65	CTX-M-15, OXA-48	CTX-M-15, OXA-48
Size, bp	107766	142552	106012	124552	185494	185434
Replicon	repA_pKPC-2	IncFIB(pQil)/ IncFII	IncFII(pHN7A8)/ IncR	IncFII/pHN7A8/ IncR	IncL/M/IncFII/IncFIA(HI1)	IncL/M/IncFII/IncFIA(HI1)
OmpK35/OmpK36	WT/WT	WT/WT	OmpK35(IS)/ OmpK36GD	OmpK35(IS)/ OmpK36GD	OmpK35(IS)/OmpK36(IS)	OmpK35(IS)/OmpK36(IS)
Other acquired AMR genes	*bla*_TEM-1D_	*aac* (3)-IIa; *ant*(2”)-Ia; *catB3*; *sul1*; *dfrA14*; *bla*_TEM-1D_	*aadA2*; *rmtB*; *fosA3*; *qnrS1*; *sul2*; *tet*(A); *dfrA14*; *bla*_TEM-1D_; *bla*_LAP-2_	*aadA2*; *rmtB*; *fosA3*; *qnrS1*; *sul2*; *tet*(A); *dfrA14*; *bla*_TEM-1D_; *bla*_LAP-2_	*aac*(6')-Ib-cr; *strA*; *strB*; *qnrB1*; *catB4*; *sul2*; *tet*(A); *dfrA14*; *bla*_TEM-1D_; *bla*_OXA-1_	*aac*(6')-Ib-cr; *strA*; *strB*; *qnrB1*; *catB4*; *sul2*; *tet*(A); *dfrA14*; *bla*_TEM-1D_; *bla*_OXA-1_
Other AMR-related mutations	GyrA-87G	None	GyrA-83I, 87G; ParC-80I	GyrA-83I,87G; ParC-80I	MgrB-62% (frameshift); GyrA-83F	MgrB-62% (frameshift); GyrA-83F
Hypervirulent plasmid size, Kb (BLASTn identity, coverage to pK2044)	229.8 (99%, 97%)	223.7 (99%, 98%)	217.8 (99%, 92%)	218.6 (99%, 92%)	164.4 (99%, 67%)	185.4 (99%, 67%)
Virulence genes	*iuc*, *iro*, *rmpADC*(Δ*rmpAC*), Δ*rmpA2*, Δ*peg-344*, *ybt*, *clb*	*iuc*, *iro*, *rmpADC*, Δ*rmpA2*, *peg-344*, *ybt*, *clb*	*iuc*, Δ*iro*, *rmpADC*, Δ*rmpA2*, *peg-344*, *ybt*	*iuc*, Δ*iro*, *rmpADC*(Δ*rmpA*), Δ*rmpA2*, *peg-344*, *ybt*	*iuc*, Δ*rmpA2*, *ybt*	*iuc*, Δ*rmpA2*, *ybt*
CPS mutation	Δ*ugd*	Δ*cpsACP*	None	None	None	None

*AMR, antimicrobial resistance; BLAST, https://blast.ncbi.nlm.nih.gov; CPS, capsule polysaccharide; ESBL, extended-spectrum β-lactamase; MLST, multilocus sequence type; OmpK35(IS), OmpK35 interrupted by insertion sequence; OmpK36GD, OmpK36 Gly134-Asp135 (GD) insertion variant; ST, sequence type; WT, wild-type.

The ST23 pVir-CRKP isolates harbored *bla*_KPC-2_ on a repA_KPC-2 plasmid (ARLG-4622) or on a pQIL-like IncFII_K2_ plasmid (ARLG-3254). The ST893 isolates encoded extended-spectrum β-lactamase (ESBL) CTX-M-15 and carbapenemase OXA-48 on a complex recombinant plasmid with IncL/M, IncFII and IncFIA(HI1) replicons. Of note, truncations in the chromosomal colistin resistance associated gene *mgrB* and insertion sequence (IS) interruptions of porins (*ompK35* and *ompK36*) were detected in both ST893 isolates. Although the ST11 isolates were carbapenem resistant (Table 3), no carbapenemase was identified. Further analysis revealed that the ST11 isolates contain the ESBL gene *bla*_CTX-M-65_, nonfunctional OmpK35 (IS interruption), and OmpK36 (glycine-aspartate insertion) (Table 2). In addition, we detected other antimicrobial resistance genes, including *fosA3*, *qnrS1*, *sul2*, and *dfrA14*, on ESBL or carbapenemase plasmids.

**Table 3:** Antimicrobial resistance of isolates in study of carbapenem-resistant, virulence plasmid–harboring *Klebsiella pneumoniae* isolates*,* United States*

Drug	Isolate					
	ARLG-4622	ARLG-3254	ARLG-4744	ARLG-7683	ARLG-3484	ARLG-4720
Aztreonam	R	R	R	R	R	R
Ceftriaxone	R	R	R	R	R	R
Ceftazidime	R	R	R	R	R	R
Cefiderocol	S	S	S	ND	S	S
Ertapenem	R	R	R	R	R	R
Imipenem	R	R	S	S	R	R
Meropenem	R	R	S	S	R	R
Ciprofloxacin	R	S	R	R	R	R
Levofloxacin	R	S	R	R	R	R
Fosfomycin	S	S	R	R	S	R
Trimethoprim/sulfamethoxazole	S	R	R	R	R	R
Colistin	I	I	I	I	R	R
Piperacillin/tazobactam	R	R	R	I	R	R
Meropenem/vaborbactam	S	S	S	S	R	R
Ceftazdimie/avibactam	S	S	S	S	S	S

*I, intermediate; ND, not determined; R, resistant; S, susceptible.

The pVir-CRKP isolates displayed multidrug resistance; their resistance genotype correlated with phenotypic resistance. For example, the ST23 and ST893 isolates encoding carbapenemase were resistant to carbapenems and cephalosporins, whereas ST11 isolates with ESBL and porin disruptions were resistant to ertapenem and cephalosporins but susceptible to imipenem and meropenem. The pVir-CRKP isolates with QRDR (*gyrA* and *parC*) mutations and quinolone resistance genes (*qnrS1* and *qnrB1*) exhibited resistance to quinolones. The ST11 pVir-CRKP isolates contained *fosA3* and were resistant to fosfomycin; the ST893 isolates with MgrB disruptions were resistant to colistin (Table 3). All isolates exhibited susceptibility to ceftazidime/avibactam and cefiderocol.

**Figure 2:**
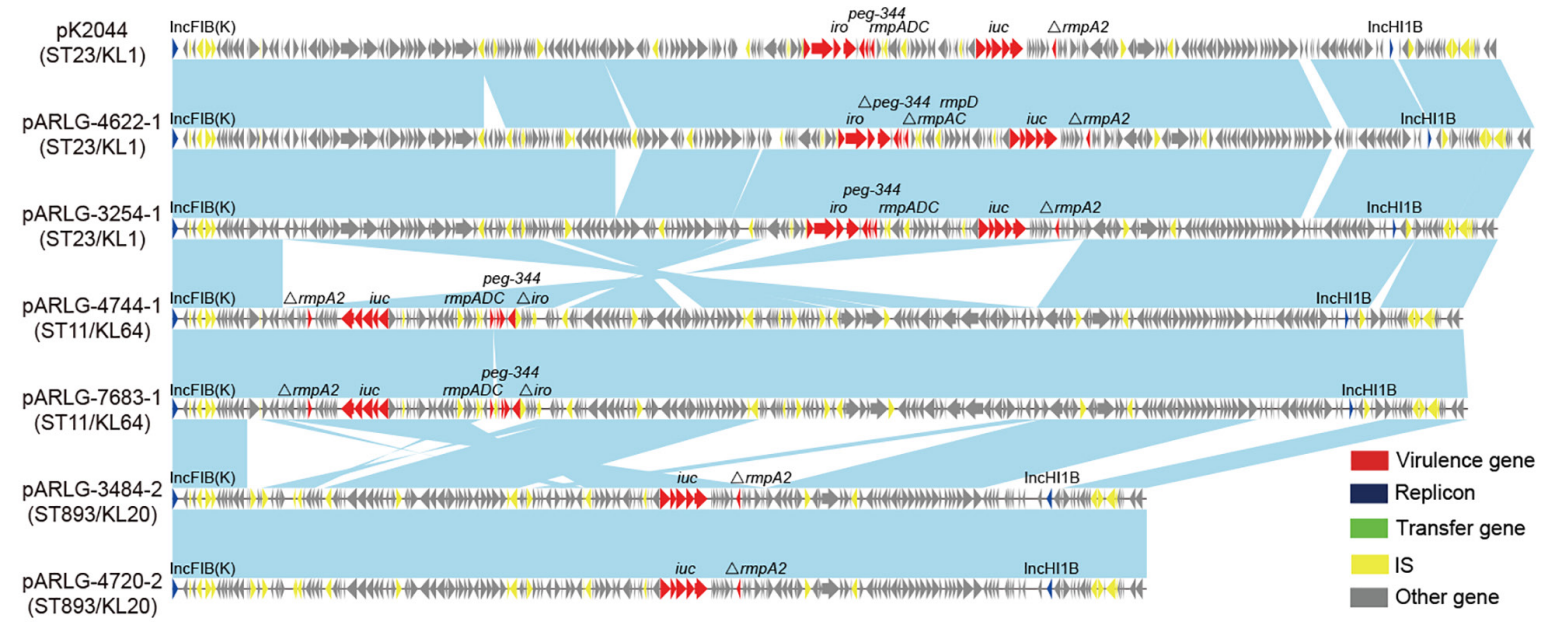
Linear alignment of virulence plasmids in isolates of carbapenem-resistant *Klebsiella pneumoniae* and hypervirulent *K. pneumoniae* NTUH-K2044*,* United States. Arrowheads represent genes; colors indicate gene function classification. Light blue shading represents regions of homology. IS, insertion sequence; KL, capsular locus; ST, sequence type.

The size of pVir in the 6 pVir-CRKP isolates was 185.4–229.8 Kb (Figure 2). The pVir in ST23 isolates (ARLG-4622 and ARLG-3254) had high sequence similarity (>99% BLAST identity and >97% coverage) to the hypervirulence plasmid pK2044 (GenBank accession no. AP006726.1) and contained intact operons encoding *iro* (*iroBCDN*) and *iuc* (*iutA-iucABCD*) and a truncated *rmpA2*. The *rmpA* operon (*rmpADC*) was intact in ARLG-3254; however, most of the operon was truncated in ARLG-4622 (except for *rmpD*). pVir in ST11 isolates (ARLG-4744 and ARLG-7683) also displayed high sequence similarity (>99% BLAST identity and ≈92% coverage) to pK2044 and contained *iuc* and *rmpDC* but had truncated *iro* and *rmpA2*. The *rmpA* locus was intact in ARLG-4744 but was interrupted by an IS in ARLG-7683. In contrast, the pVir from ST893 isolates (ARLG-3484 and ARLG-4720) showed lower similarity (>99% BLAST identity and ≈67% coverage) to pK2044 with intact *iuc* and truncated *rmpA2*; the *iro* and *rmpADC* loci were lost. All virulence plasmids possessed IncFIB(K) and IncHI1B replicons.

### Mucoviscosity and Capsule Production

**Figure 3:**
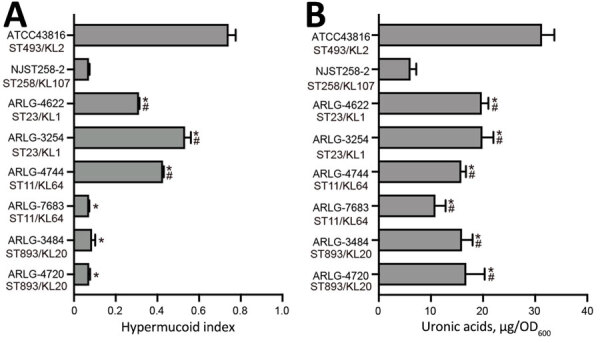
Capsule heterogeneity in 6 clinical isolates and reference isolates in study of carbapenem-resistant, virulence plasmid–harboring *Klebsiella pneumoniae* isolates*,* United States. A) Hypermucoid index scores; B) capsule production measured by uronic acid content of isolates displaying different mucoidity. The hypermucoid index and uronic acid production of the 6 isolates were compared to those of ATCC 43816 and NJST 258–2 using *t*-tests. Asterisk (*) indicates p<0.05 vs. ATCC43816; hash mark (#) indicates p<0.05 vs. NJST258_2. Results represent the mean (bars) + SDs (error bars) of 3 samples for each isolate. KL, capsular locus; OD_600_, optical density at 600 nm; ST, sequence type.

We next assessed mucoviscosity and CPS production with the 6 pVir-CRKP isolates and correlated the results with the presence of the *rmpADC* operon. The hypermucoid index for all the pVir-CRKP isolates was significantly lower than that for the reference strain ATCC43816. Isolates with intact *rmpADC* (ARLG-3254 and ARLG-4744) exhibited significantly higher mucoid indices than isolates with a truncated *rmpADC* (ARLG-4622 and ARLG-7683) or those missing *rmpADC* (ARLG-3484 and ARLG-4720). For example, the hypermucoid index for ARLG-3254 (0.53 + 0.03) was significantly greater than that for ARLG-4622 (0.31 + 0.01) (p<0.01) (Figure 3, panel A).

CPS production and mucoviscosity are 2 different properties of *K. pneumoniae* with overlapping and confounding phenotypes (*30*). In our study, CPS production was independent of the presence of intact *rmpADC* or *rmpA2*, and correlated with each isolate’s genetic background and capsule type, because ST23-KL1 isolates produced the greatest amount of CPS (19.84 + 1.65 µg/OD_600_), followed by ST893-KL20 isolates (16.40 + 2.74 µg/OD_600_), and ST11-KL64 isolates (13.36 + 2.96 µg/OD_600_) (Figure 3, panel B).

### Differential Survival of pVir-CRKP Isolates 

**Figure 4:**
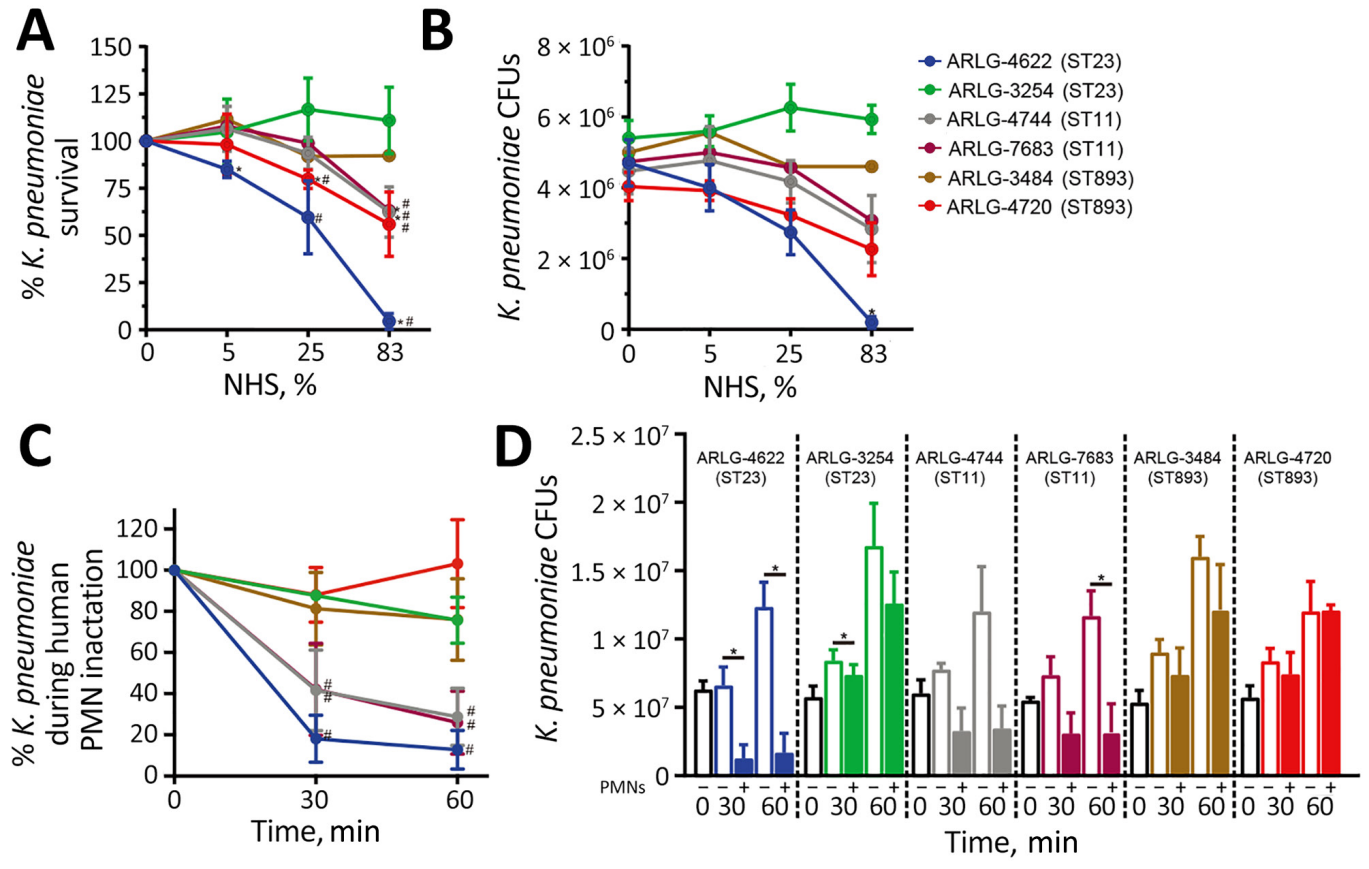
Serum and PMN bactericidal activity in 6 clinical isolates in study of carbapenem-resistant, virulence plasmid–harboring *Klebsiella pneumoniae* isolates*,* United States. A–B) The indicated clinical isolates were cultured with 0%, 5%, 25%, or 83% NHS for 1 hour, and recovered viable bacteria were enumerated as both percentages (A) and absolute values (B). Asterisk (*) indicates p<0.05 vs. 0% serum by using a repeated-measures analysis of variance and Dunnett posttest; hash mark (#) indicates p<0.05 vs. ARLG-3254 in 25% or 83% NHS by using a *t*-test. C–D) Survival of clinical isolates determined following synchronized PMN phagocytosis assays at 30 or 60 min as described. Data are expressed as percentage survival (C) or colony-forming units (D). Results represent the mean (dots) + SD (error bars) of 3 separate experiments. Asterisk (*) indicates p<0.05 as determined by using a repeated-measures analysis of variance and Bonferroni posttest (selected pairs of data) or Kruskal-Wallis test and Dunn posttest for nonparametric analysis of ARLG-4744; hash mark (#) indicates p<0.05 vs. ARLG-3254 for 30 or 60 min in PMN by using a *t*-test. We conducted statistical analyses by using CFUs. NHS, normal human serum; PMN, polymorphonuclear neutrophils; ST, sequence type.

To assess the ability of the US pVir-CRKP isolates to circumvent killing by the human innate immune system, we tested survival of the 6 selected clinical isolates in NHS. Our previous study demonstrated that clinical *K. pneumoniae* strains without pVir usually have <40% survival in 25% NHS and <20% survival in 83% NHS after 1 hour (*26*). We found that the pVir-CRKP isolates (except for ARLG-4622) had >75% (96.1% + 16.0%) survival in 25% NHS and >50% (76.9% + 24.9%) survival in 83% NHS after 1 hour (Figure 4, panels A, B), indicating those isolates have enhanced capacity to survive in NHS by comparison.

Although the pVir-CRKP isolates (except ARLG-4622) displayed greater survival in NHS, they exhibited varied survival during phagocytic interaction with human PMNs (Figure 4, panels C, D). For example, survival of ARLG-3254 (ST23), ARLG-3484 (ST893) and ARLG-4720 (ST893) was not notably lower after 60 min of phagocytic interaction with PMNs. By comparison, survival of ARLG-4744 (ST11) and ARLG-7683 (ST11) decreased significantly by interaction with PMNs (29% + 14% vs. 26% + 15%; p<0.05).

### Virulence of pVir-CRKP Isolates in Mouse Pneumonia Model

**Figure 5:**
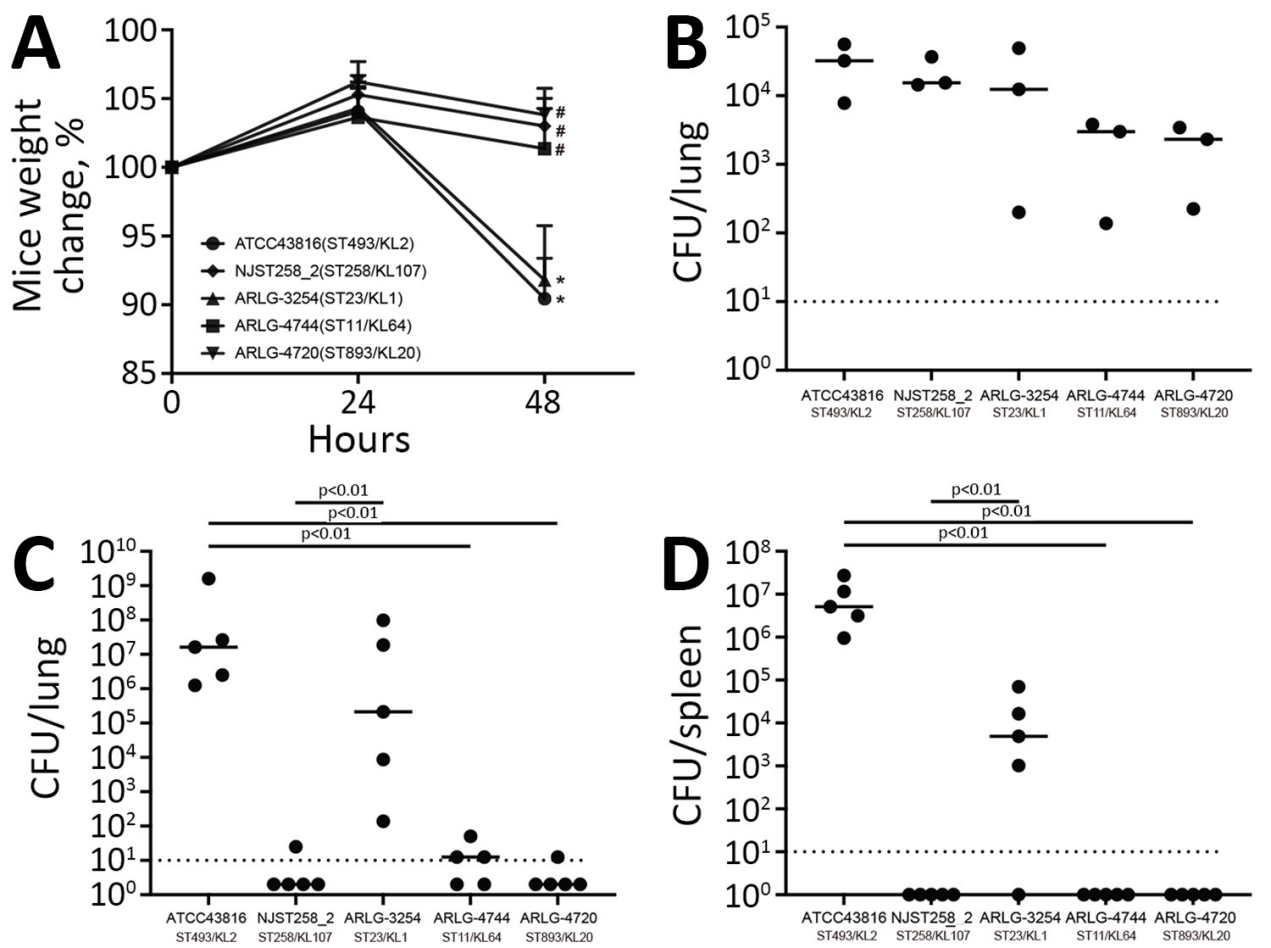
Virulence comparison of 3 selected isolates in study of carbapenem-resistant, virulence plasmid–harboring *Klebsiella pneumoniae* isolates*,* United States. Virulence phenotypes were determined in a murine model of *K. pneumoniae* pneumonia. A) Changes in mouse weight relative to the baseline at 0 h during the course of infection (n = 5). Results represent the mean (dots) + standard deviation (t bars) of the indicated number of samples. Asterisks (*) indicate p<0.05 vs. 24 h; hash marks (#) indicate p<0.05 vs. ATCC43816. B–D) Bacteria recovered from mouse lungs at 2 h (B, n = 3) and 48 h (C, n = 5) postinfection and from spleens at 48 h postinfection (D, n = 5). Individual CFU values (dots) and mean values (horizontal lines) are shown. We used *t*-tests for all the comparisons. Dashed lines indicate limits of detection. KL, capsular locus; ST, sequence type.

We evaluated the in vivo virulence of pVir-CRKP using a mouse pneumonia model. Weight of the mice (measured by weight relative to the 0 h baseline) inoculated with HvKP strains, ATCC43816 (virulent control strain) and the ARLG-3254 (ST23) clinical isolate, was significantly reduced (p<0.01) compared with weight of mice inoculated with a clinical *K. pneumoniae* ST258 control strain (NJST258_2) or the other isolates tested (Figure 5, panel A). At 2 hours postinfection, we observed no significant difference in viable bacteria recovered from the lungs and spleens of mice inoculated with 3 pVir-CRKP isolates and 2 control strains (Figure 5, panel B). However, at 48 hours postinfection, the bacterial load in the lungs (Figure 5, panel C) and spleens (Figure 5, panel D) in mice infected with ATCC43816 and ARLG-3254 was significantly higher (p<0.01) than that in mice infected with NJST258_2, ARLG-4744 and ARLG-4720, suggesting ARLG-3254 (ST23) has significantly higher virulence in vivo than NJST258_2 and 2 other pVir-CRKP isolates at this inoculum.

### Phylogenetic Analyses of pVir-CRKP Isolates

To track the possible origin of US pVir-CRKP isolates, we performed phylogenetic analyses with additional genomes of the same ST from the National Center for Biotechnology Information Genome database (https://www.ncbi.nlm.nih.gov/datasets/genome). We aligned the top 50 genetically related genomes and the ARLG-4622 genome sequence to the ST23 reference genome ARLG-3254 (GenBank accession no. CP067777), identifying 5,037 core-genome SNPs (cgSNPs). The ARLG-3254 and ARLG-4622 differed by 280 pairwise cgSNPs (Appendix Figure 1). ARLG-3254 (from Houston, Texas, USA) was most closely related to the ST23 strains isolated in the United States, whereas ARLG-4622 (from Cleveland, Ohio, USA) was more similar to strains reported in China (Appendix Figure 1). Both ST23 isolates showed long phylogenetic distance from the first KL1-ST23 pVir-CRKP strain reported in the United States (*5*) (Appendix Figure 1). For the ST11 strains, the top 50 genetically related genomes and the ARLG-7683 genome sequence were aligned to the reference genome ARLG-4744 (GenBank accession no. CP139237); we identified 1,023 cgSNPs. The ARLG-4744 and ARLG-7683 collected in Atlanta were genetically similar (differing by 11 pairwise cgSNPs) and most closely related to strains collected in China (Appendix Figure 2). For the ST893 strains, all 18 publicly available genomes and the ARLG-3484 genome sequence were aligned to the ST893 reference genome ARLG-4720 (GenBank accession no. CP139225); we detected 1,466 cgSNPs. The ARLG-4720 and ARLG-3484 from Atlanta differed by only 6 pairwise cgSNPs and were most similar to strains collected in Iran in 2018 (Appendix Figure 3).

## Discussion

In this study, we analyzed the genomic features of 884 CRKP isolates from CRACKLE-2 study US cohorts and identified 6 (0.7%) pVir-CRKP isolates. The 6 pVir-CRKP isolates belonged to 3 different STs, and each isolate exhibited enhanced production of CPS, survival in NHS and resistance to PMN killing compared with the well-characterized and virulence plasmid–lacking classical *K. pneumoniae* strain NJST258_2 (the predominant CRKP clone in the United States). Phylogenetic analyses revealed that 5 of 6 US pVir-CRKP isolates were genetically similar to strains reported from China and Iran.

pVir-CRKP strains have mostly been reported from East Asia. Here, we report the emergence of pVir-CRKP strains in the United States during 2016–2017. The first reported US pVir-CRKP strain (DHQP1701672) was collected in 2016 from a 65-year-old patient after travel to South America (*5*). DHQP1701672 is an ST23 strain harboring pVir and pKpQIL-like *bla*_KPC-2_ plasmid. Kochan et al. (*31*) screened the genomes of 2,608 multidrug-resistant *K. pneumoniae* isolates from 3 different US studies and identified 47 isolates containing >1 HvKP virulence gene. In this study, we identified 2 ST23 pVir-CRKP isolates; 1 harbored a pKpQIL-like *bla*_KPC-2_ plasmid. However, we noted significant phylogenetic distance between those isolates and DHQP1701672, indicating that they did not evolve from DHQP1701672. One ST23 pVir-CRKP isolate showed close genetic distance to an HvKP strain reported in the United States. Given that this pVir-CRKP isolate harbored a pKpQIL-like *bla*_KPC-2_ plasmid, which is prevalent in North America (*32*), we hypothesize that the isolate may have arisen from the acquisition of a pKpQIL-like *bla*_KPC-2_ plasmid by a local US ST23 HvKP. Another ST23 pVir-CRKP isolate showed close phylogenetic distance to the strains reported in China. In addition, we identified ST11 and ST893 pVir-CRKP isolates. ST11 is the most common clone of CRKP and pVir-CRKP in China (*9*), and the 2 ST11 pVir-CRKP isolates identified here were phylogenetically similar to strains from China. Last, ST893 is a minor clone mainly reported in China (*33*) and Iran (*34*), and the ST893 pVir-CRKP isolates reported in our study were similar to strains reported from Iran. Overall, our study indicates that, although most US pVir-CRKP isolates were genetically related to strains from other countries, they may also arise domestically through the acquisition of a prevalent circulating carbapenemase gene.

Ex vivo experiments showed that most of the pVir-CRKP isolates displayed increased antiserum capacity in NHS and high antiphagocytosis capacity toward PMNs. Although the ST23 pVir-CRKP isolate ARLG-4622 harbored the 5 virulence genes on pVir (*13*), it exhibited remarkably reduced antiserum and antiphagocytosis capacities. Further genomic analysis revealed that *rmpAC* and *ugd* genes were truncated in ARLG-4622. *RmpA* amplifies capsule production and mucoviscosity in HvKP strains, thereby enhancing their resistance to neutrophil phagocytosis and killing mediated by serum complement (*11*). *Ugd* plays a crucial role in the formation of an antiphagocytic capsule, which serves to protect bacteria from the host’s immune system (*35*). The defects of *RmpA* and *Ugd* potentially reduced the virulence of ARLG-4622, making it more vulnerable to NHS and PMNs. Therefore, it is crucial to assess loss-of-function mutations in virulence genes and CPS synthesis genes when attempting to genetically predict the virulence of *K. pneumoniae*.

Because our understanding of *K. pneumoniae* virulence, especially that of multidrug-resistant HvKP strains, is limited, predicting virulence remains a challenge and warrants further investigation. In the mouse pneumonia model, only the ST23 pVir-CRKP isolate demonstrated enhanced virulence; that finding is consistent with the results from a study that found most *K. pneumoniae* isolates with both multidrug-resistance and virulence genes had low virulence (*31*). Although ST11 and ST893 pVir-CRKP isolates did not demonstrate enhanced virulence in the mouse in vivo, the enhanced ability of those isolates to avoid killing by human serum and PMNs may confer enhanced survival during human interaction. Such a phenotype could pose a substantial risk to immunocompromised populations, potentially leading to fatal outcomes (*8*). For example, in our study, the patient infected with ARLG-4744 (ST11) had a high Pitt score and died within 30 days.

A limitation of our study is that the incidence of pVir-CRKP in the United States could be underestimated because we collected only CRKP isolates from hospitalized patients and did not include intestinal colonization samples; nonetheless, a strength of our study is that we used a standardized, contemporary approach to include hospitalized patients with CRKP. Second, although we identified potential clonal transmission within ST11 and ST893 pVir-CRKP isolates, we do not have enough information, especially information regarding environmental samples, to confirm the transmission events. Third, the small sample size of pVir-CRKP–infected patients may limit the accuracy of the observed mortality rate. Finally, the data were somewhat outdated, and a more recent dataset could be more clinically relevant.

In summary, pVir-CRKP with enhanced virulence and carbapenem resistance has emerged in the United States. pVir-CRKP strains exhibited heterogeneous in vitro, ex vivo, and in vivo virulence, warranting further investigation to genetically predict their pathogenicity. Isolates identified in this study were predominantly genetically related to strains from other countries, which highlights the critical importance of global molecular surveillance. 

Appendix Additional information about carbapenem-resistant, virulence plasmid–harboring *Klebsiella pneumoniae*, United States.
