# Supplementary material for: Carbapenem-Resistant, Virulence Plasmid–Harboring Klebsiella pneumoniae, United States
Source: Emerg Infect Dis. 2025 Apr;31(4):761–71. doi: 10.3201/eid3104.241396 (PMC11950267; doi:10.3201/eid3104.241396)
Supplement: Appendix — Additional information about carbapenem-resistant, virulence plasmid–harboring Klebsiella pneumoniae, United States. [file 24-1396-Techapp-s1.pdf]

Article DOI: https://doi.org/10.3201/eid3104.241396

*EID cannot ensure accessibility for supplementary materials supplied by authors. Readers who have difficulty accessing supplementary content should contact the authors for assistance.*

# Carbapenem-Resistant, Virulence Plasmid–Harboring *Klebsiella pneumoniae,* United States

## Appendix

## Supplementary Methods

### Microbiology

Bacterial identification and susceptibility testing were performed by each contributing microbiology laboratory using Biotyper (Bruker, Billerica, MA, USA), MicroScan (Beckman Coulter, Atlanta, GA, USA), or VitekMS, Vitek2, or Etest (all bioMérieux, Durham, NC, USA), BD Phoenix or BBL disks (both BD, Durham, NC, USA), Sensititer (Thermo Fisher, Waltham, MA, USA), or disk diffusion methods. Carbapenem resistance was determined based on the Clinical and Laboratory Standards Institute (CLSI) interpretive criteria for meropenem or imipenem nonsusceptibility (MIC, ≥4 µg/ml).

### Quantification of Mucoviscosity and Capsule Production

Overnight cultures were adjusted to an $OD_{600}$ of 1 in PBS. The suspensions were then centrifuged and the $OD_{600}$ values (hypermucoid index) of the supernatant were measured in triplicate.

The capsule was quantified using the uronic acid method. Overnight cultures were adjusted to an $OD_{600}$ of 0.2, incubated for 6 hours and the $OD_{600}$ was determined. Capsule was extracted with 1% Zwittergent 3–14. Galacturonic acid was used as a standard. The samples

were next treated with concentrated sulfuric acid and then 3-hydroxybiphenyl was added to generate the chromophore and $OD_{520}$ values were measured. Uronic acids were quantified by comparing the OD with the galacturonic acid standard curve.

**Serum Bactericidal Activity and Polymorphonuclear Neutrophil (PMN) Phagocytosis Assays**

Venous blood and/or heparinized venous blood were obtained from healthy individuals in accordance with a protocol approved by the IRB for Human Subjects at the National Institutes of Health (protocol 01IN055). Subjects provided informed consent before participation.

Serum bactericidal activity was determined using a published method (*1*). $5\times10^5$ bacteria (in 50 µl RPMI 1640 medium buffered with 10 mm Hepes (RPMI/H) were combined with normal human serum (NHS) and RPMI/H in an assay vol of 300 µl. Assay tubes were rotated for 1 h and aliquots of the assay were diluted serially and plated on LB agar plates. Colony forming units (CFUs) were enumerated the following day. Percent survival was calculated with $CFU_{with\ NHS}/CFU_{without\ NHS}\times100$.

Human PMNs were isolated using a standard method (*2*). A published assay was used to determine PMN killing (*1*). Bacteria ($2.5\times10^6$), PMNs ($5\times10^5$ in RPMI/H), and NHS were combined in 24-well plates (444 µl final assay vol) that had been precoated with 20% NHS. Assay plates were centrifuged and then transferred to a $CO_2$ incubator for 1 h. Plates were transferred to ice and 0.1% saponin was added to lyse PMNs for 15 min. An aliquot of each assay mixture was diluted in saline and plated on LB agar. Colonies were enumerated the following day.

**Animal Infection Models**

A well-established murine intranasal infection model was used for this study (*3*). Female BALB/c mice (8 each group) aged 6–8 weeks were anesthetized by intraperitoneal injection with a mixture of ketamine (100 mg/kg) and xylazine (20 mg/kg). A total of 50 µl of *K. pneumoniae* suspension (~$10^4$ CFUs) was placed on the nares of mice (25 µl per nare) to allow for aspiration

into the lungs. At 2 h (n = 3) and 48 h (n = 5) post-infection, mice were euthanized and lungs and spleens were collected immediately (within 5 min of euthanasia). For bacterial burden analysis, tissues were added to an equal volume of PBS (100 mL PBS/100 mg tissue) and processed using a tissue homogenizer. Bacterial burdens of each tissue were quantified by serial dilutions on LB agar plates. *K. pneumoniae* strains ATCC43816 (ST493/KL2) and NJST258–2 (ST258/KL107) were used as controls for comparison. The weights were observed for all mice at 24 h and 48 h post-infection. All vertebrate animal experiments were performed with the approval of HMH Institutional Animal Care and Use Committee.

**Phylogenetic Analysis**

Additional genome and metadata from 85,944 publicly available *K. pneumoniae* genomes were collected from the NCBI RefSeq database (accessed in May 2023). MLSTs were determined using mlst v2.22 (github.com/tseemann/mlst), identifying 644 ST23, 5,208 ST11, and 18 ST893 genomes. Using mash distance (*4*), the 50 genetically closest genomes or all the genomes (if less than 50) were selected for each ST for further phylogenetic analyses. Parsnp v1.7.4 (*5*) was then used to construct phylogenetic trees based on all the selected assemblies. ARLG-3254 (GenBank: CP067777), ARLG-4744 (GenBank: CP139237) and ARLG-4720 (GenBank: CP139225) were used as references in the tree constructions. Core genome SNPs (cgSNPs) were called by Snippy v4.6.0 (github.com/tseemann/snippy).

**Supplementary References**

1. DeLeo FR, Porter AR, Kobayashi SD, Freedman B, Hao M, Jiang J, et al. Interaction of multidrug-resistant hypervirulent *Klebsiella pneumoniae* with components of human innate host defense. MBio. 2023;14:e0194923. PubMed https://doi.org/10.1128/mbio.01949-23

2. Kobayashi SD, Voyich JM, Buhl CL, Stahl RM, DeLeo FR. Global changes in gene expression by human polymorphonuclear leukocytes during receptor-mediated phagocytosis: cell fate is regulated at the level of gene expression. Proc Natl Acad Sci U S A. 2002;99:6901–6. PubMed https://doi.org/10.1073/pnas.092148299

3. Lawlor MS, Hsu J, Rick PD, Miller VL. Identification of *Klebsiella pneumoniae* virulence determinants using an intranasal infection model. Mol Microbiol. 2005;58:1054–73. PubMed https://doi.org/10.1111/j.1365-2958.2005.04918.x

4. Ondov BD, Treangen TJ, Melsted P, Mallonee AB, Bergman NH, Koren S, et al. Mash: fast genome and metagenome distance estimation using MinHash. Genome Biol. 2016;17:132. PubMed https://doi.org/10.1186/s13059-016-0997-x

5. Treangen TJ, Ondov BD, Koren S, Phillippy AM. The Harvest suite for rapid core-genome alignment and visualization of thousands of intraspecific microbial genomes. Genome Biol. 2014;15:524. PubMed https://doi.org/10.1186/s13059-014-0524-x

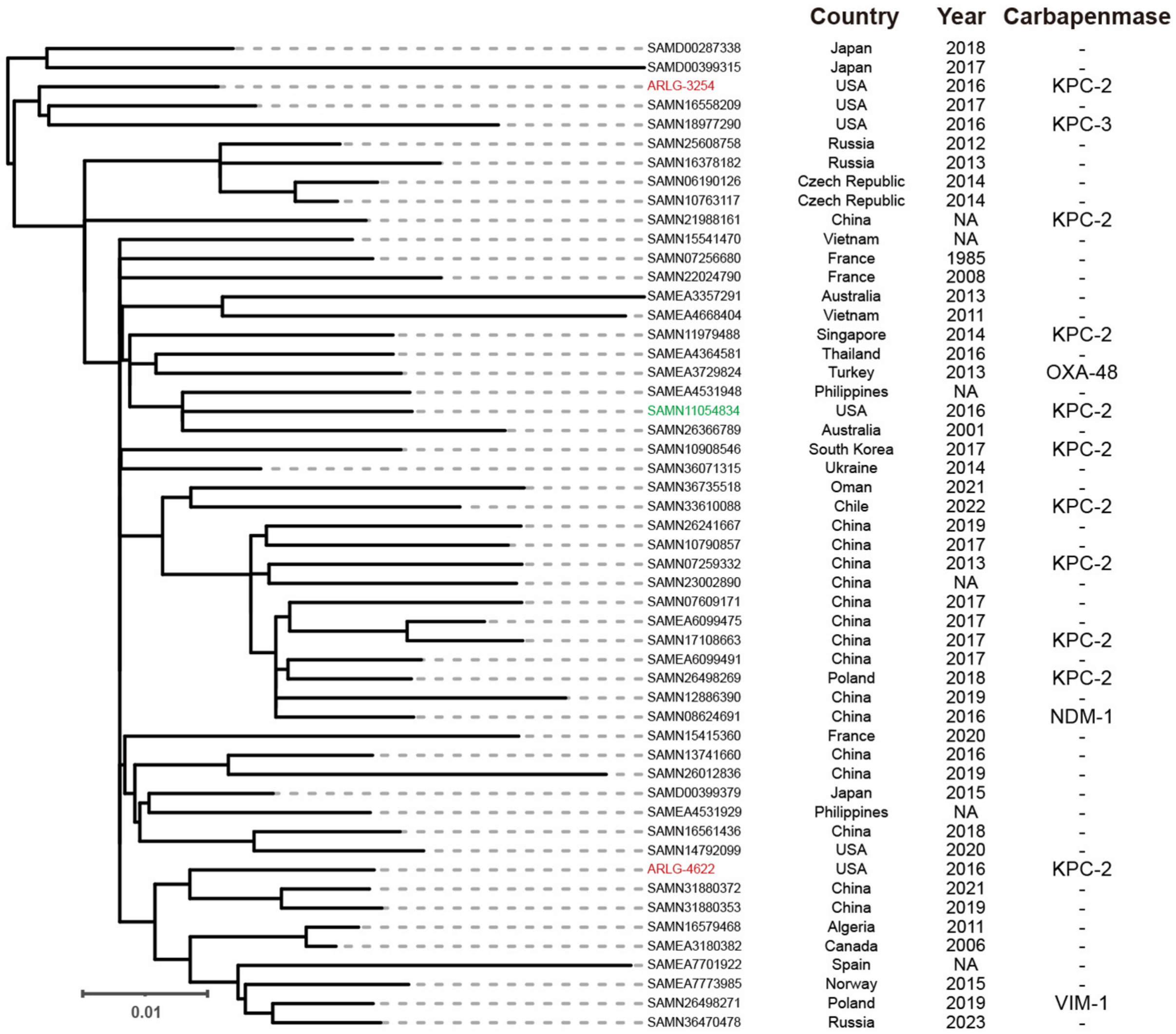


**Appendix Figure 1.** Phylogenetic analysis of ST23 *K. pneumoniae* isolates. Names in red are pVir-CRKP isolates from this study. Name in green is the first KL1-ST23 pVir-CRKP strain DHQP1701672 reported in the United States.

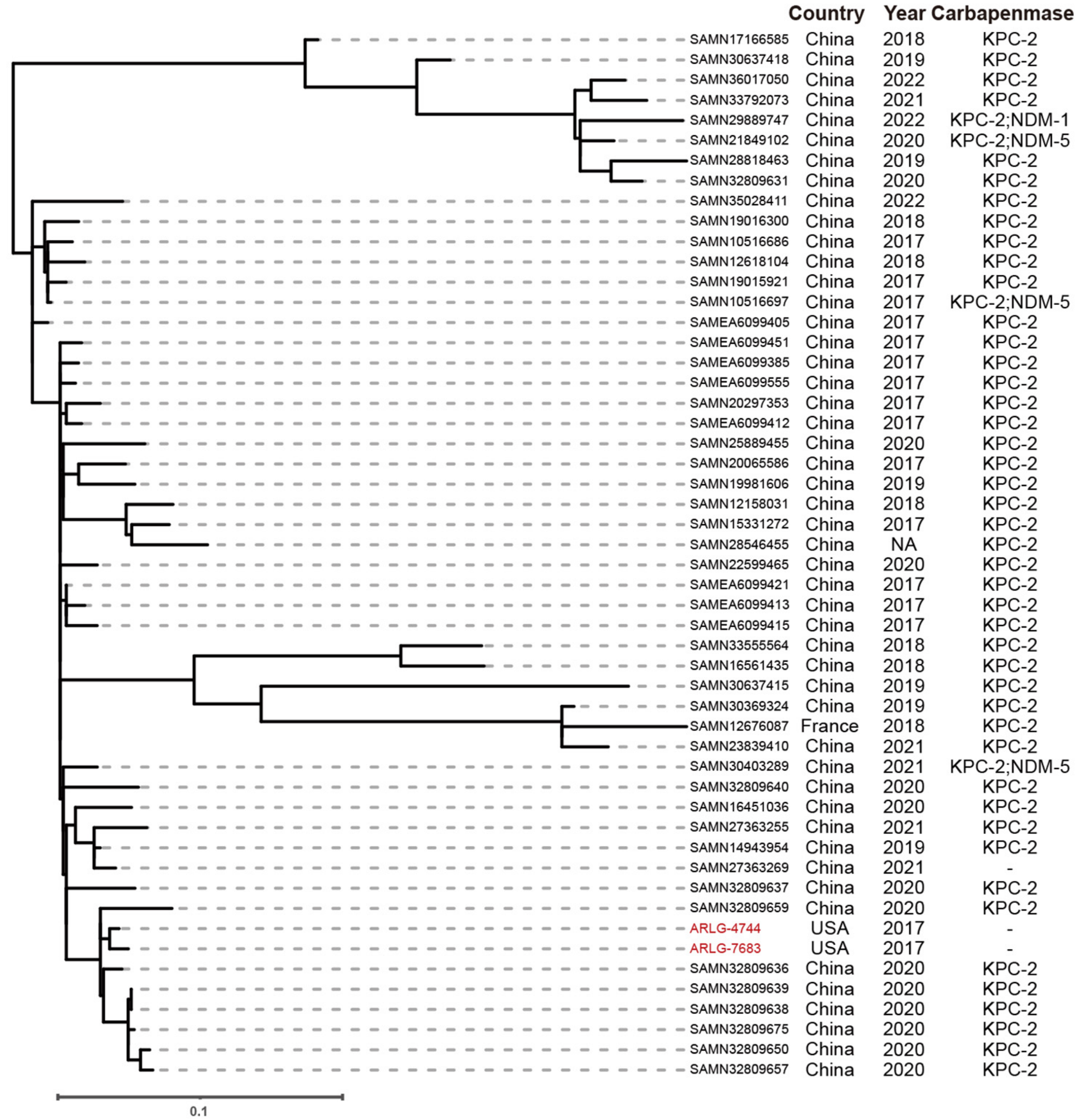


**Appendix Figure 2.** Phylogenetic analysis of ST11 *K. pneumoniae* isolates. Names in red are pVir-CRKP isolates from this study.

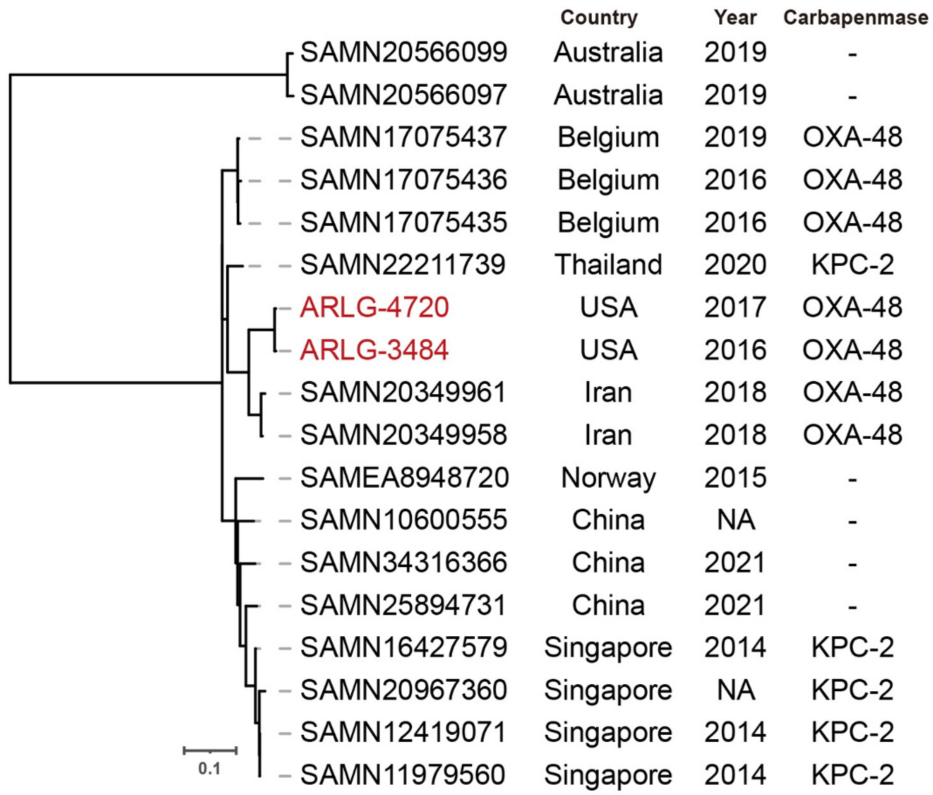


**Appendix Figure 3.** Phylogenetic analysis of ST893 *K. pneumoniae* isolates. Names in red are pVir-CRKP isolates from this study.
